# The Unfortunate

**Published:** 1872-02

**Authors:** 


### THE UNFORTUNATE.

There are one hundred thousand persons in the United States who are either blind, insane, or idiotic. Two great practical and suggestive facts present themselves in the specifications of these statistics. The proportion of negroes among the unfortunates is very much the larger, showing the deplorable results of ignorance and degradation.

Again, there are more men among all classes of unfortunates, except among the insane; in this calamity women are in the majority, and it is very greatly to be deplored; every noble heart bleeds at the contemplation of such a sad, sad reality; should it be my wife, my daughter, my sister, my mother, what an agony! The causes of

INSANITY AMONG WOMEN

are not hidden, and they are removable. They are mainly two.

1. Anxiety about making a living.
2. The circumstances of the married relation.

The palpable and effectual remedy for the former is to open up to them additional avenues of maintenance.

Certain changes in the marriage relation are a necessity.

Both remedies are in the hands of parents. All radical changes in society come from above downwards; if we will elevate the race, every parent under the United States government, every intelligent Christian parent especially, should educate every daughter of the family to some handicraft or calling, and make them adepts in it, which would yield them a support in case they were never married; or if married, were left widows, unprovided for. In addition, every husband should feel himself bound to provide for his wife first, and for each daughter, as he is able, a sufficient sum of money, to yield them, as interest, at least one hundred dollars, the principal to go to some one else at their death, the interest to be inalienable by any act of theirs, and to be paid to them in person, quarterly, during their lives. By this arrangement, the principal could never be touched by any act of the wife, and the interest would be certain to be paid at short intervals, without the possibility of alienation to husband, brother, or any one else. It is a small sum, to be sure, but a small amount of money certainly paid at the day, by simply presenting the demand at the United States treasury, would be a very great comfort. There is many a woman in the United States at this moment, whose feelings would be ecstatic if assurance were given that for the remainder of life she should receive twenty-five dollars every three months, without fail or defalcation. Many a woman did we say? that does not tell the one hundredth part of the tale; there are such women who have lived in their own mansions, have ridden in their own carriages, who have delighted in their own country-seats, and spent many a summer at the sea-side and the springs. Only a few months ago a lady said to me, "All that my husband can get to eat for breakfast is a single roll of bread. We lived in Washington Square a few years ago, and owned our own country-seat." Old New Yorkers can count on their fingers over and over again, giving to each one at each count a different name, just such histories. A man near our office occupies a room nine by twelve, and gets a dinner wherever he can find it — often fishes for an invitation to a lunch, from some friend of earlier days. At one time he had some money paid to him, and did not know what to do with it, and finally had it deposited to his credit in a Connecticut bank, — seventy-three thousand dollars!

There are tens of thousands of men all over the country who could, without missing it, place to the credit of wife and each daughter, a thousand dollars in United States bonds, in such a way that such wife and daughter could not, if they would, retransfer it.

Others could give large sums, and thus put the loved ones beyond destitution forever. But men are so much afraid of reducing their capital in trade, or are so anxious to employ every dollar they can rake and scrape in the hope of making more, and have a larger amount to leave their families, that they cannot persuade themselves to such a course. They believe it is not necessary, that they have too much money to make such a course important, that there is no danger of their failure; and in many cases the feeling is that it is too trifling a sum for "my wife," for "my daughter." But if all who are now able to make such provision for their wives and daughters were to do so, and all who follow after would do so, as soon as they were able, many a rich man's daughter would be saved from beggary, within thirty years, many a rich man's wife from an insane asylum.

There are many ways in which daughters could be brought up to make their own living; and our State legislature should come to their aid, and the general government also. The post-offices of our country should be opened to them, as also all offices which require mainly writing, copying, keeping accounts, and other clerical occupations; almost all in-door employments ought to be appropriated to them, which do not involve heavy work, — retail stores, shops, telegraph offices; and they could be profitably employed to some extent at our banks and banking houses. Multitudes of them, by being properly trained to nurse the sick, would be angels of mercy at the bedside, and have liberal compensation besides. Good nursing will cure three fourths of the ordinary ailments of humanity, without any medicine. Women may with propriety be editors, and writers, and authors; a woman can be a good nurse; but to make of her a doctor, a lawyer, or a clergyman, is a dishonor and a degradation. They are always and everywhere, now and forevermore, by their physical, mental, and moral nature, wholly unfitted and incompetent to these places.

It was said that the circumstances of the married relation tended to promote insanity in women. In what the change should consist is a most difficult problem; it is earnestly hoped that it may be worked out in such a way as to add to domestic happiness, and elevate women to a higher plane in the counsels and rights of the household. There is trouble on both sides. Physicians, more than all others, have opportunities of knowledge in this direction their observation is that men are too often inconsiderate, unsympathetic. Woman always pines for sympathy; with it she can endure more than a man; without it, she fades like a flower without water, or chafes behind the bars of her prison house, until she falls into madness. It is from the farm-house that most such women come, because they are over-taxed, and without its being intended, are treated more like hirelings or beasts of burden. Let farmer husbands, and all others, look to this.

